# Spectral computer-tomography and the ability to detect occult femoral neck and scaphoid fractures – A systematic review and exploratory meta-analysis

**DOI:** 10.1016/j.ejro.2025.100686

**Published:** 2025-09-24

**Authors:** Camilla Thøgersen Buxbom, Amalie Braithwaite, Søren Hess, Lars Lykke Hermansen, Martin Weber Kusk

**Affiliations:** a Department of Orthopedics, University Hospital of Southern Denmark, Esbjerg, Denmark; b Department of Radiology, University Hospital of Southern Denmark, Esbjerg, Denmark; c Department of Nuclear Medicine, Odense University Hospital, Odense, Denmark; d Department of Clinical Research, Faculty of Health Sciences, University of Southern Denmark, Odense, Denmark; e Department of Regional Health Research, Faculty of Health Sciences, University of Southern Denmark, Odense, Denmark

**Keywords:** Femoral neck, Scaphoid bone, Spectral CT, DECT, Photon-counting detector technology, Occult fractures

## Abstract

**Objective:**

This systematic review aims to describe the ability of spectral computed tomography (SCT) to identify bone marrow oedema (BME) in the scaphoid bone and the femoral neck compared to magnetic resonance images (MRI).

**Materials and methods:**

PubMed, Embase and Cochrane Library were searched from the 1st of October 2024; eligible studies had patients over 15 years of age, underwent SCT and had MRI as a gold standard. Sensitivities, specificities, negative predictive values (NPV) and positive predictive values (PPV) were noted or calculated from available information. Bias and applicability were assessed using QUADAS-2 tools. A random-effects model was used in the meta-analysis, and heterogeneity was assessed using I^2^ statistics.

**Results:**

1061 studies were identified and screened by title/abstract. Twenty-two studies underwent full-text assessment. A total of four were included, of which three were suitable for meta-analysis regarding the scaphoid bone. Pooled sensitivity was 93 %, specificity was 98 %, PPV was 92 % and NPV was 98 %. A single study concerning the femoral neck was located, with sensitivity, specificity, PPV and NPV of 87 %, 94 %, 93 % and 89 % respectively.

**Conclusions:**

Data regarding the ability of SCT to detect BME in the femoral neck and scaphoid bone are promising, but limited, with only small studies available. There is a need for larger prospective studies, regarding both the detection of occult fractures in the femoral neck and the scaphoid bone.

## Highlights

• Spectral CT shows promising potential in detecting occult scaphoid fractures.
• The use of Spectral CT for detecting occult scaphoid fractures may reduce extra follow-ups and lower hospital costs.
• The application of Spectral CT for femoral neck fractures remains unclear.

## 1 Introduction

The majority of bone fractures are diagnosed by plain radiography. However, a low-energy trauma may result in a non-displaced fracture, which can be difficult to detect with conventional X-ray imaging, potentially resulting in overlooked or occult fractures. Two of the most clinically important occult fractures are scaphoid and femoral neck fractures (FNF) [1]. Scaphoid fractures constitute 60 % of carpal fractures, up to 22 % [2] of the primary X-ray examinations of scaphoid fractures are false negatives and therefore not treated accordingly [3]. 60 % of proximal femoral fractures are FNF, and 3–10 % of these are estimated to be occult [4]. The potential consequences of missing these fractures can be substantial, leading to complications such as chronic pain, impaired mobility, avascular necrosis and long-term disability non-union [5], [6].

The gold standard for diagnosing an occult fracture is magnetic resonance imaging (MRI). However, MRI capacity varies largely among hospitals and is not as accessible as X-ray or Computed tomography (CT), influencing local guidelines regarding the detection of occult fractures [7]. Furthermore, MRI is subject to absolute and relative contraindications, e.g. metal or electronic implants, claustrophobia and obesity. Thus, MRI is not used as the first-line modality, which can lead to delayed diagnosis and incorrect treatment, with higher risk of long-term complications.

Spectral computed tomography (SCT) is a common term for CT techniques utilising the variation in tissue attenuation response at different photon energies. This can be achieved either by exposing the patient to different tube voltages, either with Dual Energy CT (DECT) or rapid voltage switching. Alternatively, energies can be separated on the detector side, either using Dual Layer Detector (DLCT) or, more recently, photon-counting detector (PCD) technology [8]. The latter also offers substantially improved cortical and trabecular detail compared to previous CT systems, potentially offering higher diagnostic accuracy [9].

SCT virtual non-calcium (VnCa) images can be used to visualize bone marrow edema (BME), which can potentially be used for better detection of bone contusions and occult fractures [6], [10], [11]. Furthermore, compared to MRI, CT has higher isotropic spatial resolution, and conventional, morphological CT images can be evaluated in addition to VnCa images from the same acquisition, while effective radiation doses in extremity scans have been steadily reduced [12]. Thus, given that CT is a fast and widely available examination, with very few contraindications, suggests its’ potential applicability in ruling out radiographically occult fractures in an acute setting.

The emphasis of prior research has primarily focused on the usage of SCT to detect fractures in specific areas, such as the vertebrae, knee and ankle joints [13], [14], [15], making it necessary to expand the investigation to the scaphoid and hip areas, due to the large population effected each year [5].

SCT is still not routinely used in hospitals in Denmark for the diagnosis of occult fractures, and to our knowledge, no systematic reviews regarding the diagnostic accuracy of SCT in detecting occult femoral neck and scaphoid fractures have been performed. This review and exploratory meta-analysis aims to provide an overview of published evidence quantifying the diagnostic accuracy of SCT in detecting suspected occult femoral neck and scaphoid bone fractures, compared to MRI as the gold standard.

## 2 Material and methods

### 2.1 Design

This study was designed as a systematic review and the protocol was registered in the PROSPERO database before the screening of studies began (Prospero ID: CRD42024594669).

### 2.2 Eligibility criteria

We included studies of patients over the age of 15, with SCT performed on suspicion of radiographically occult fractures in the femoral neck and/or scaphoid bone.

Acceptable gold standard was MRI, including relevant (STIR or equivalent) sequences for bone marrow oedema (BME) visualization.

The following article types were excluded:

- Case reports, letters, conference proceedings, technical reports and animal studies.
- Studies in other language than English, Danish, German, Swedish and Norwegian.
- Articles where sensitivity and specificity were not reported or did not contain data, allowing for self-calculation.
- Studies published before the year 2000 as the first clinically available SCT scanner was not available before 2006 [16], but to ensure inclusion of any potential studies performed on prototype scanners.

### 2.3 Information sources and search strategy

PubMed, Embase and Cochrane Library were searched the 1st of October 2024, search string can be found in Supplementary material 1. Furthermore, the reference lists of all included articles in the full-text screening were reviewed for relevant papers. The systematic review was conducted according to the PRISMA guidelines [17].

### 2.4 Selection process and data collection process

Covidence (Covidence PLC, Australia) was used for screening of articles. Duplicates were removed and non-relevant studies excluded during screening. A title/abstract screening was followed by full-text assessment performed independently by AB, CB and MK, to decide whether a study met the inclusion criteria. Quantitative data were extracted from included studies by two independent reviewers AB and CB.

### 2.5 Data items

For each study, sensitivity, specificity and positive/negative values (PPV/NPV) were recorded or calculated from the reported 2 × 2 contigency tables of index- vs. gold standard test results.

### 2.6 Study risk of bias assessment

Risk of bias was assessed with QUADAS-2 [18]. The assessment was done by study level. Two researchers (AB and CB) evaluated the risk of bias assessment tool individually. In cases of disagreement, the matter was discussed until consensus was reached. However, this was not necessary, as the two researchers (AB and CB) were in agreement throughout.

### 2.7 Synthesis methods

A meta-analysis was performed comparing diagnostic accuracy measures of BME on SCT vs MRI. In case of multiple reviewers within a study, the mean was calculated and used.

**Fig. 1:**
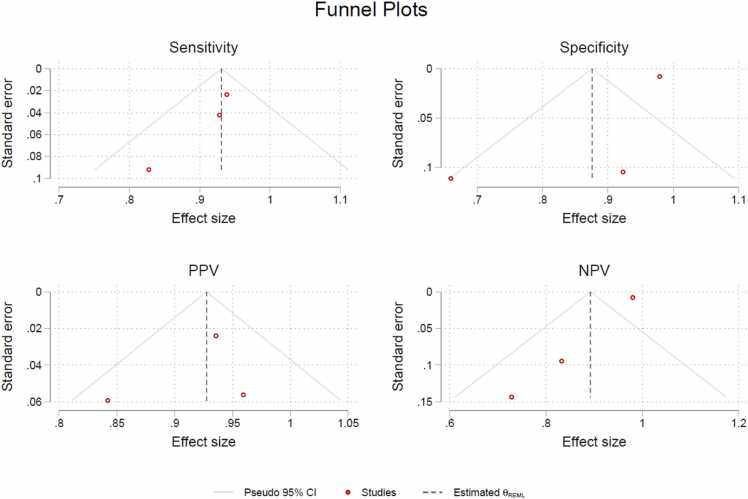
Funnel plots of diagnostic measures of the included studies on scaphoid fractures.

**Fig. 2:**
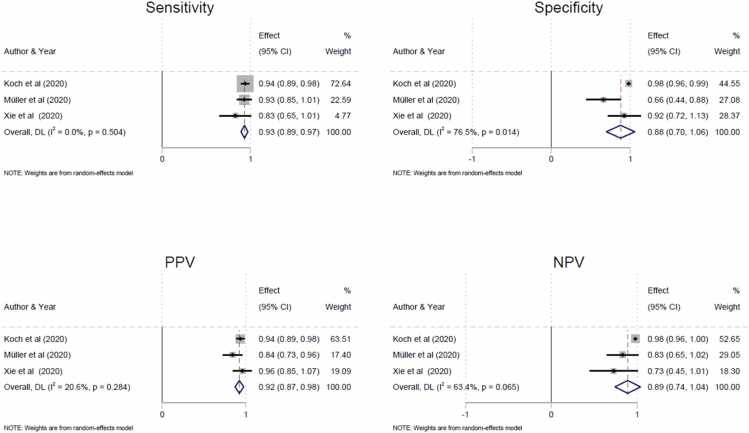
Forest plots of diagnostic measures of the included studies on scaphoid fractures, with pooled results and associated 95 % confidence intervals.

Consistency among the studies was calculated by a heterogeneity test (*I*^*2*^) [19]. Funnel plots were produced to assess the risk of publication bias in the meta-analysis (Fig. 1). We used forest plots to assess the certainty in the body of evidence for our outcome (Fig. 2).

We are aware that both I^2^ and funnel plots are less reliable when fewer than 10 studies are included. Therefore we would like to emphasize that the meta-analysis results from these are exploratory only.

To avoid issues with division by zero and to ensure that all cells have a value that can be used for calculations, 0.5 has been added to all cells in the 2 × 2 table of Xie et al. [20], as zero false positive results was reported (modified Haldane-Anscombe correction [21]). We applied a random-effects model for meta-analysis of diagnostic test accuracy data and conducted all statistical analysis in STATA 18 (StataCorp, USA).

## 3 Results

### 3.1 Study selection

**Fig. 3:**
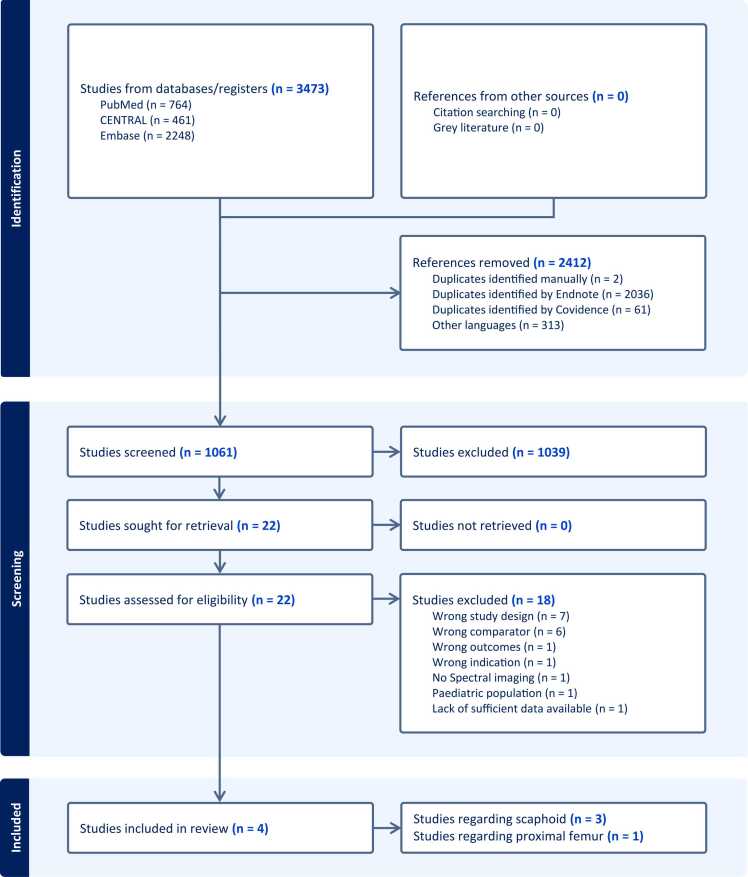
Flowchart of study search and inclusion process.

A total of 3160 studies were identified across the three databases, and after removal of duplicates, 1061 studies remained for title/abstract screening. We performed full-text screening of 22 studies and finally included four studies (Fig. 3). Three studies focused on the scaphoid bone [20], [22], [23], and only one study on the proximal femur [24].

The details of the included studies and the quality assessment are shown in Table 1, Table 2, respectively. Furthermore, the pooled sensitivities, specificities, NPV and PPV are shown in Fig. 2. For further details regarding QUADAS-2, we refer to Supplementary material 2. Information regarding the technical parameters of SCT can be seen in Supplementary material 3.

**Table 1:** Characteristics of the included studies.

Author	Anatomical location	Study design and inclusion period	No. patients *(Male/Female)*	Mean age (Range)	Time between Spectral CT and reference standard (days)	Blinding	No. readers	Reader experience (years)	TP	FP	FN	TN
Koch et al.^a^ (2020)	Scaphoid	Retrospective Jan. 2017–Dec. 2019	141 *(74/67)*	43 years *(19–80 years)*	1 day (range 0–2 days)	Yes	6 radiologists	8/7/6/6/5/5	438	6	18	384
Müller et al.^a^ (2020)	Scaphoid	Prospective Jan. 2018– Nov. 2018	50 *(24/22)*	47 years *(18–85 years)*	Within 24 h	Yes	4 radiologists	11/10/2/2	43	2	17	138
Xie et al. a, b (2020)	Scaphoid	Prospective NR	20 *(12/8)*	51 years *(20–76 years)*	6 days (range 0–9 days)	Yes	2 radiologists	NR	23.5	0.5	5.5	12.5
Schierenbeck et al. (2023)	Proximal femur	Retrospective 2019–2022	32 *(NR)*	NR	NR	Yes	2 radiologists	5/15	NR	NR	NR	NR

TP = True positive, FP = False positive, FN = False negative, TN = True negative, NR = Not Registered.

a Data reported under TP, FP, FN and TN are combined results for all readers in the respective study.

b ""modified Haldane-Anscombe"" (mHA) correction, 0.5 has been added, due to zero false positive results.

**Table 2:** QUADAS-2.

	Risk of bias					Applicability concerns		
Study	Patient selection	Index test	Reference standard	Flow and timing		Patient selection	Index test	Reference standard
Koch et al. (2020)	Low risk	Low risk	Low risk	Low risk		Low risk	Low risk	Low risk
Müller et al. (2020)	Low risk	Low risk	Low risk	Low risk		Low risk	Low risk	Low risk
Xie et al. (2020)	Low risk	Low risk	Low risk	Unclear risk		Low risk	Low risk	Low risk
Schierenbeck et al. (2023)	High risk	Unclear risk	Low risk	Unclear risk		Low risk	Low risk	Low risk

One study was excluded due to lack of sufficient data. In this study, all the carpal bones were grouped together, and individual results for each bone were not reported [25]. The authors were contacted regarding Supplementary data without success.

### 3.2 Scaphoid

#### 3.2.1 Study characteristics

Koch et al. [22] retrospectively investigated 141 consecutive patients with acute wrist trauma. Mean interval between SCT and MRI was one day (range 0–2 days). All SCT results were evaluated by six radiologists blinded to the clinical assessment and MRI results. For BME detection, they found a sensitivity of 96 %, specificity of 99 %, PPV of 99 %, NPV of 96 % and accuracy of 97 % [22].

Müller et al. [23] prospectively evaluated 50 wrists in 46 consecutive patients with wrist trauma and clinical suspicion of a wrist fracture. Mean interval between SCT and MRI was less than 24 h. All SCT results were evaluated by four radiologists who were blinded to clinical information and MRI. They found a sensitivity of 72 %, specificity of 99 %, PPV of 96 %, NPV of 89 % and accuracy of 91 % [23].

The study by Xie et al. [20] prospectively investigated 20 consecutive patients with a suspected occult scaphoid fracture. The mean interval between MRI and SCT was six days (range 0–9 days). Two radiologists blinded to MRI findings evaluated the SCT images. They found a sensitivity of 82 %, specificity of 100 %, PPV of 100 %, NPV of 71 % and accuracy of 88 % [20].

### 3.3 Femoral neck

Schierenbeck et al. [24] retrospectively evaluated 32 consecutive patients with and without BME. Firstly, patients with extremity fractures were identified, and control patients without any fractures of the extremities were included. Two radiologists blinded to clinical assessment evaluated MRI and SCT images. They found a sensitivity of 86.7 %, specificity of 94.1 %, PPV of 92.9 %, NPV of 88.9 % and AUC of 90.6 % [24].

A meta-analysis of the proximal femur will not be conducted due to the lack of additional articles.

## 4 Discussion

The use of SCT for BME detection is still a relatively new imaging technique, as evidenced by the relatively low number of articles included in this systematic review. In the studies regarding the scaphoid fractures, SCT demonstrated high pooled sensitivity, specificity, PPV, and NPV, with values at 93 %, 98 %, 92 %, and 98 %, respectively, in detecting scaphoid fractures following normal X-ray; however, even though these results appear promising, these findings should be considered exploratory due to the limited number of studies, and interpreted with caution. We only identified a single study with a specific focus on FNF. This may be due to the fact that virtual non-calcium (VnCa) images are susceptible to beam-hardening and photon starvation artefacts [26], especially in larger patients, which are more likely to occur in the pelvic region and/or with metal implants in the contralateral hip. Also, the wrist or knee is more easily centered within the limited dual energy field of view in SCT systems. This is not always possible with the femoral neck, especially in patients in severe pain.

SCT has long been considered a promising new imaging technique, becoming more widely available during the last 10–15 years. However, the applicability is still not well-established due to limited knowledge and learning curves for radiologists regarding imaging interpretation [10]. SCT VnCa images can be used to visualize BME, which may potentially improve the detection of bone contusions and occult fractures [6], [10], [11].

Furthermore, compared to MRI, CT has higher isotropic spatial resolution, and conventional, morphological CT images can be evaluated in addition to VnCa images from the same acquisition, while effective radiation doses in extremity scans have been steadily reduced [12]. Thus, given that CT is a fast and widely available examination with very few contraindications, suggests its' potential applicability in ruling out radiographically occult fractures in an acute setting.

A potential limitation in implementing new imaging techniques is associated with a learning curve that may involve potential variability and error margins. The two readers in the study of Müller et al. [23] only had two months of experience each in musculoskeletal radiology. However, we do not believe that our results are affected by this. In a study by Fukuda et al. [27], they found that even though readers had different levels of experience, the inter-reader agreement was high, suggesting that all radiologists can confidently identify BME.

Although the included studies were assessed as having a low risk of bias, and the results of this review and meta-analysis are therefore considered reliable, they should still be interpreted with caution due to the limited number of studies included, as bias could have been introduced unintentionally. Two of the included studies [20], [23] are prospective studies, and both presented lower sensitivities compared to the retrospective study [22], which can indicate selection bias in the retrospective study. The nearly equal representation of male and females included in the study, as illustrated in Table 1, helps to reduce the risk of gender-related bias. All biases has been attempted to keep at a minimum, which is why we conducted the comprehensive searches across multiple databases and assessed publication bias using a funnel plot. Additionally, all included studies has a wide age range, demonstrating its applicability in both the younger and older populations.

AI tools for fracture detection on planar radiographs are increasingly implemented in Danish hospitals. However, there is currently limited evidence for their performance in scaphoid fracture detection [28]. This may, in part, be attributed the challenges in obtaining training data reflecting the large variations in projections and acquisition techniques [29].

A recent study [30] showed a detection rate of 41 % for radiographically occult fractures using a deep learning (DL) network, which is inferior to those demonstrated above.

On the contrary, it is conceivable that the combination of BME visualization and high spatial resolution of CT with AI for fracture detection may further improve the diagnostic accuracy, also for non-specialist clinicians in the future.

As mentioned earlier, previous research on SCT in the musculoskeletal area has primarily focused on detecting fractures in the vertebrae, knee and ankle joints [13], [14], [15]. Bäcker et al. [15] reported a pooled sensitivity of 89 %, specificity of 91 % and accuracy of 89 % of SCT in detecting BME in 515 patients with vertebrae injuries compared to MRI. François et al. [31] compared SCT to MRI in 14 consecutive patients with suspected vertebral compression fractures. They reported a sensitivity of 95 %, specificity of 94 %, PPV of 79.8 % and a NPV of 98.7 %. Similar results were found in a meta-analysis from Wang et al. based on 17 studies [32], where SCT was used to detect BME in 625 patients with lower limb injuries. They reported pooled sensitivity between 80 % and 84 %, specificity ranging from 89 % to 97 %, with high diagnostic accuracy across different fractures. Our findings align well with these results and even demonstrates higher accuracy in the detection of occult scaphoid fractures. This consistency across multiple studies enhances the credibility of our results. However, these findings should be interpreted with caution due to the limited number of studies included. Therefore, further research in areas such as the scaphoid and hip could yield valuable insights. By expanding the focus of investigations, we can better understand the effectiveness of SCT in diagnosing fractures in these regions.

If a new technique, such as SCT, becomes more commonly used in daily clinical practice compared to MRI, it is essential to recognise that BME may not always be directly equivalent to a bone fracture. Its presence in an acutely injured bone only suggest a high likelihood of fracture [33]. This distinction between oedema and treatment-requiring fractures is crucial for clinicians to keep in mind when interpreting imaging results and making treatment decisions based on SCT images. Potentially, SCT can lead to over-treatment, especially if the technology detects minor BME that may not require treatment. This over-treatment can cause patient anxiety, and increase healthcare costs and resource utilisation.

Based on the three included studies, we report a pooled PPV of 92 %, which indicates that 5–10 % of patients being allocated to scaphoid fracture treatment are false positives. On the contrary, and more importantly, we report a high NPV of 98 %. Therefore, patients discharged after SCT with no fracture detected, can rely on this more confidently, as this result is completely at the level of MRI [2]. This will potentially reduce the need for additional outpatient follow-ups due to the low number of false negatives, ultimately saving hospital costs that can be used elsewhere.

Even though the presented evidence is limited, and should be verified by larger, prospective studies, SCT appears promising for detection of occult scaphoid fractures. If the reported accuracy figures can be replicated in clinical practice, SCT in the acute/subacute phase has great potential to significantly reduce immobilization time and cost, similar to the benefits of early MRI [34]. The short examination time, wide availability, minimal patient discomfort and contraindications, combined with a very low radiation dose speaks in favor of implementing this modality. For hip fractures we suggest awaiting further studies before recommending routine SCT for occult FNF. This examination imparts significantly higher radiation doses and is more susceptible to degradation from e.g. contralateral metal implants.

It is important to strike a balance between ensuring that fractures requiring treatment are not missed, while avoiding overtreatment of benign conditions. As demonstrated by Xie et al. [20], optimal fracture detection requires inspection of both VnCa images, as well as morphological images, e.g. to detect cortical defects. Given the high spatial resolution achievable in PCD-CT, it is conceivable that VnCa images may help to direct the readers' attention to BME, thus minimising the occurrence of overlooked fracture sites [35], while inspection of morphological images may help to prove or disprove the presence of a fracture.

In conclusion, we report a promising potential regarding the future use of SCT in detecting occult scaphoid fractures, while its application on FNF remains somewhat unclear based on this systematic review. The presented results should be considered preliminary, and therefore further and larger prospective studies regarding detection of occult fractures, is needed to confirm these observations. The expected improvements in patient management should be balanced against the increased, albeit relatively small, radiation exposure imparted by CT. Further research and clinical guidelines are needed to refine the criteria for treatment decisions based on SCT findings. This could help ensure that only those patients who truly need intervention receive it, optimising patient care and resource allocation.

## CRediT authorship contribution statement

**Amalie Braithwaite:** Writing – review & editing, Writing – original draft, Visualization, Supervision, Project administration, Methodology, Investigation, Formal analysis, Data curation. **Lars Lykke Hermansen:** Writing – review & editing, Methodology, Conceptualization. **Søren Hess:** Writing – review & editing. **Martin Weber Kusk:** Writing – review & editing, Validation, Software, Investigation, Formal analysis, Data curation, Conceptualization. **Camilla Thøgersen Buxbom:** Writing – review & editing, Writing – original draft, Visualization, Supervision, Project administration, Methodology, Investigation, Formal analysis, Data curation.

## Ethical Statement for Solid State Ionics

Hereby, I Camilla Thøgersen Buxbom consciously assure that for the manuscript ‘Spectral computertomography and the ability to detect occult femoral neck and scaphoid fractures – A systematic review and exploratory meta-analysis’ the following is fulfilled:

1) This material is the authors' own original work, which has not been previously published elsewhere.
2) The paper is not currently being considered for publication elsewhere.
3) The paper reflects the authors' own research and analysis in a truthful and complete manner.
4) The paper properly credits the meaningful contributions of co-authors and co-researchers.
5) The results are appropriately placed in the context of prior and existing research.
6) All sources used are properly disclosed (correct citation). Literally copying of text must be indicated as such by using quotation marks and giving proper reference.
7) All authors have been personally and actively involved in substantial work leading to the paper, and will take public responsibility for its content.

The violation of the Ethical Statement rules may result in severe consequences.

To verify originality, your article may be checked by the originality detection software iThenticate. See also http://www.elsevier.com/editors/plagdetect.

I agree with the above statements and declare that this submission follows the policies of Solid State Ionics as outlined in the Guide for Authors and in the Ethical Statement.

## Declaration of Competing Interest

The authors declare that they have no known competing financial interests or personal relationships that could have appeared to influence the work reported in this paper.

## Glossary

FNF: Femoral neck fractures
MRI: Magnetic resonance imaging
CT: Computed tomography
SCT: Spectral computed tomography
DECT: Dual Energy CT
DLCT: Dual Layer Detector
PCD: Photon-counting detector
BME: Bone marrow oedema
PPV: Positive predictive values
NPV: Negative predictive values
VnCa: Virtual non-calcium
