# Supplementary material for: Spectral computer-tomography and the ability to detect occult femoral neck and scaphoid fractures – A systematic review and exploratory meta-analysis
Source: Eur J Radiol Open. 2025 Sep 24;15:100686. doi: 10.1016/j.ejro.2025.100686 (PMC12508587; doi:10.1016/j.ejro.2025.100686)
Supplement: Supplementary file 1 — Supplementary material [file mmc1.pdf]

Supplementary material

Supplementary material 1: Search string for scaphoid and femoral neck fracture

Query	Results
Search: **((((((((((((compute* tomograph*) OR (CAT)) OR (CT)) OR (MSCT)) OR (MDCT)) OR (Multislice)) OR (Multi*slice)) OR (multidetector)) OR (multi*detector)) OR (computed tomography, x ray[MeSH Terms])) AND ((((((((((Dual Energy) OR (Multi*energy)) OR (Dual source)) OR (Energy resolved)) OR (Energy based)) OR (Switching)) OR (Spectral)) OR (Dual Layer)) OR (Virtual non calcium)) OR (Photon counting))) AND ((((((((((carpal bones[MeSH Terms]) OR (wrist joint[MeSH Terms])) OR (femur[MeSH Terms])) OR (scaphoid bone[MeSH Terms])) OR (Hip)) OR (Femoral)) OR (Femur)) OR (Scaphoid)) OR (Wrist)) OR (Carpal))) AND (((Injur*) OR (Fracture*)) OR (fractures, bone[MeSH Terms])) Filters: **Danish, English, German, Norwegian, Swedish, from 2000 - 2024**	749

Supplementary material 2: QUADAS-2

	Risk of bias				Applicability concerns		
Study	Patient selection	Index test	Reference standard	Flow and timing	Patient selection	Index test	Reference standard
Koch et al. (2020)	Yes<br>Yes<br>Yes<br>Low risk	Yes<br>Yes<br>Low risk	Yes<br>Yes<br>Low risk	Yes<br>Yes<br>Yes<br>Yes<br>Low risk	Low risk	Low risk	Low risk
Müller et al. (2020)	Yes<br>Yes<br>Yes<br>Low risk	Yes<br>Yes<br>Low risk	Yes<br>Yes<br>Low risk	Yes<br>Yes<br>Yes<br>No<br>Low risk	Low risk	Low risk	Low risk
Xie et al. (2020)	Yes<br>Yes<br>Yes<br>Low risk	Yes<br>Yes<br>Low risk	Yes<br>Yes<br>Low risk	No<br>Yes<br>Yes<br>Yes<br>Unclear risk	Low risk	Low risk	Low risk
Schierenbeck et al. (2023)	Yes<br>No<br>No<br>High risk	Yes<br>Unclear<br>Unclear risk	Yes<br>Yes<br>Low risk	Unclear<br>Yes<br>Yes<br>No<br>Unclear risk	Low risk	Low risk	Low risk

Supplementary material 3: Technical parameters of spectral computed tomography (SCT)

	Scanner						Technical parameters			Interpretation		
Author	Vendor	Model	Technology	Collimation (mm)	Tube voltage (kVp)	Tube current (mAs)	No. image planes	Mean CTDI (mGy)	Reconstructed slice thickness/increment (mm)	Reconstrution kernel	Reconstructed images postprocessing	Outcome classification
Koch et al.	Siemens	Somatom Force	Dual Source	192 x 0.6	150(Sn)/90	180/180	3	9.4	1/0.8	Bone, soft tissure	VnCa Conventional	Binary
Müller et al.	Siemens	Somatom Force	Dual Source	128 x 0.6	150(Sn)/80	NR	3	6.17	2/1	NR	VnCa	Binary
Xie et al.	GE Medical Systems	Revolution CT	kVp switching	NR	140/70	260	NR	NR	0.625/0.625	Bone, soft tissure	VnCa Conventional	Binary
Schierenbeck et al.	Philips Healthcare	Spectral IQON	Dual Layer Detector	64 x 0.625	120	NR	NR	NR	3	NR	Conventional	Binary

NR = Not registered, VnCa = Virtual NonCalcium
